# [GeRu_6_(CO)_18_HI]: A Germanium‐Centered Ruthenium Carbonyl Cluster with Aromatic Ring Current

**DOI:** 10.1002/advs.202309043

**Published:** 2024-03-21

**Authors:** Silke Wolf, Ralf Köppe, Jens Treptow, Wolfram Feuerstein, Jonas Wenzel, Frank Breher, Peter W. Roesky, Florian Weigend, Wim Klopper, Claus Feldmann

**Affiliations:** ^1^ Institute for Inorganic Chemistry Karlsruhe Institute of Technology (KIT) Engesserstraße 15 D‐76131 Karlsruhe Germany; ^2^ Fachbereich Chemie Philipps‐Universität Marburg Hans‐Meerwein‐Straße 4 D‐35043 Marburg Germany; ^3^ Institute of Physical Chemistry Karlsruhe Institute of Technology (KIT) Fritz‐Haber‐Weg 2 D‐76131 Karlsruhe Germany

**Keywords:** germanium‐centered, liquid‐phase synthesis, ruthenium carbonyl cluster, sigma aromaticity

## Abstract

The carbonyl cluster compound [GeRu_6_(CO)_18_HI] is unique in regard to its structure and bonding with a GeRu_6_ cluster core, a planar GeRu_4_HI unit, extensive multi‐center bonding, and an aromatic ring current similar to benzene (9‐10 nA T^−1^). The open‐shell cluster core is a Ge‐centered five‐membered Ru_4_(Ru_2_) ring with CO ligands and an additional H and I atom, each bridging two Ru atoms on opposite sides of the cluster core. The compound is prepared at 130 °C in a weakly‐coordinating ionic liquid.

## Graphical abstract

A complex bonding situation characterizes the novel carbonyl cluster compound [GeRu_6_(CO)_18_HI] with a Ge‐centered five‐membered Ru_4_(Ru_2_) cluster core, extensive multi‐center bonding, a planar GeRu_4_HI unit, and an aromatic ring current similar to benzene.

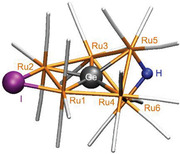

Aromaticity is usually the domain of organic chemistry and originates from delocalized π‐electrons.^[^ ^1^ ^]^ In recent years, however, aromaticity was also found for cluster compounds showing delocalized π‐electrons or delocalized σ‐electrons.^[^ ^2^ ^]^ Selected examples comprise cluster cores such as {Bi_6_}, [Ge_24_]^4–^, {Th_3_}, [Th@Bi_12_]^4–^, [Zn_6_Ge_16_]^4–^, [Au_2_Sb_16_]^4–^ or [Zn_3_]^+^.^[^ ^3^ ^]^ Generally, cluster compounds with aromatic features either contain only a few metal atoms (≤4) in a planar arrangement (e.g., triangular *M_3_* or rectangular *M_4_* systems),^[^ ^2^, ^3^ ^]^ or they exhibit sphere‐like multi‐atom systems (e.g., [Ge_24_]^4–^, [Th@Bi_12_]^4–^, [Zn_6_Ge_16_]^4–^, [Au_2_Sb_16_]^4–^).^[^ ^3a,b,d‐f^ ^]^ Moreover, the cluster core is usually coordinated and stabilized by π‐ligands (e.g., C_5_H_5_, C_5_Me_5_, C_8_H_8_). The aromaticity is typically indicated by a specific ring current and ranges at values of 5–30 nA T^−1^.^[^ ^4^ ^]^ Although metal carbonyls represent an important class of cluster compounds, σ‐aromaticity with an aromatic ring current was yet described only for rare examples (i.e., closed‐shell carbonyl cluster anions [Bi_6_Mo_3_(CO)_9_]^4–^ and [(CrGe_9_)Cr_2_(CO)_13_]^4–^).^[^ ^5^ ^]^

Cluster compounds in the Ru–Ge system are well‐known, in principle.^[^ ^6^ ^]^ Larger Ru–Ge clusters most often exhibit trigonal‐bipyramidal, octahedral, or square‐pyramidal cluster cores.^[^ ^6a‐c^ ^]^ [Ru_5_(CO)_11_(µ‐GePh_2_)_4_(µ_5_‐C)], for instance, contains a square‐pyramidal Ru_5_ unit with four Ru–Ru edges of the square bridged by GePh_2_ units.^[^ ^6a^ ^]^ [(µ_3_‐Ge{Ru(CO)_2_(µ_5_‐C_5_Me_4_H)})_2_Ru_3_(CO)_9_] contains a trigonal‐pyramidal Ru_3_Ge_2_ core with Ge atoms at the pyramid tips bond to an additional Ru atom.^[^ ^6b^ ^]^ [Ru_5_(CO)_12_(µ_3_‐Ge^t^Bu)_2_(µ_4_‐Ge^t^Bu)(H)] contains a Ru_5_Ge octahedron with two faces capped by additional Ge atoms.^[^ ^6c^ ^]^ [Ru_4_(µ_4_‐GePh)_2_(µ‐GePh_2_)_4_(CO)_8_], finally, exhibits a Ru_4_Ge_2_ octahedron with all Ru–Ru bonds bridged by additional GePh_2_ units.^[^ ^7^ ^]^ Ruthenium clusters with small atoms enclosed are also well‐known (e.g., H, C) and result in square‐pyramidal or octahedral building units (e.g., [Ru_6_C(CO)_17_], [Ru_5_(CO)_11_(µ‐GePh_2_)_4_(µ_5_‐C)], [{PtRu_5_(CO)_13_‐(P^t^Bu_3_)}(µ‐H)_3_(GePh_3_)(µ_5_‐C)]).^[^ ^6^, ^8^ ^]^ Despite this generally rich chemistry, Ru cluster compounds enclosing heavier elements and/or aromaticity–to the best of our knowledge–have not been reported, so far.

In the following, we report on the non‐charged ruthenium carbonyl cluster [GeRu_6_(CO)_18_HI] with an unusual open‐shell GeRu_6_ cluster core (**Figure** **1**). Herein, a Ru_4_(Ru_2_) ring is centered by Ge and coordinated by 18 CO ligands as well as by one hydrogen and one iodine atom. A five‐membered Ru_4_(Ru_2_) ring centered by a heavy element like Ge is new and characterized by an unusual bonding situation with a substantial σ‐aromatic ring current.

**Figure 1:**
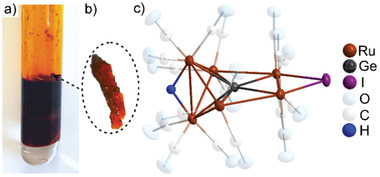
Scheme illustrating the synthesis of [GeRu_6_(CO)_18_HI]: a) Sealed glass ampoule after reaction, b) exemplary red‐colored crystal, c) the molecular structure of [GeRu_6_(CO)_18_HI].

[GeRu_6_(CO)_18_HI] was prepared by reaction of GeI_4_ and Ru_3_(CO)_12_ in [BMIm][OTf] (OTf: triflate) as ionic liquid at 130 °C in argon‐filled glass ampoules (Figure 1a). The synthesis can be rationalized based on the following equation (Figure S1, Supporting Information): GeI_4_ + 3 Ru_3_(CO)_12_ + [C_8_H_15_N_2_]^+^ → [GeRu_6_(CO)_18_HI] + [Ru(CO)_4_]_n_ + C_8_H_14_N_2_ + 2 Ru^2+^ + 3 I^–^ + 14 CO. Hydrogen was obviously transferred from the cation of the ionic liquid, which was described before in the literature.^[^ ^9^ ^]^

The title compound crystallizes with orange to red crystals in the monoclinic space group *P*2_1_ */c* and was prepared with a yield of about 20% (Figure 1b; Table S1, Supporting Information). The composition was validated by energy‐dispersive X‐ray spectroscopy (EDXS) in regard to the ratio of the heavy elements to be Ru:Ge:I = 6.4(3):1.1(2):1.0(2) (expected:6:1:1; in regard of hydrogen see MS and FT‐IR below). [Ru(CO)_4_]_n_ was obtained as a side product and identified by EDXS and X‐ray powder diffraction (Figure S1, Supporting Information).^[^ ^10^ ^]^ As a black powder, moreover, [Ru(CO)_4_]_n_ can be easily distinguished from the red crystals of the title compound.

[GeRu_6_(CO)_18_HI] exhibits a Ru_6_Ge cluster core surrounded by 18 carbonyl ligands, one hydrogen atom, and one iodine atom (Figure 1c; Figures S2–S4, Supporting Information). In detail, the compound can be designated as [(µ_6_‐Ge){Ru(CO)_3_}_6_(µ_2_‐H)(µ_2_‐I)]. The germanium atom is centered in a five‐membered Ru_4_(Ru_2_) ring and solely coordinated by Ru atoms. The split five‐membered Ru_4_(Ru_2_) ring shows a planar arrangement of four Ru atoms (Ru1 to Ru4) and the central Ge. The additional Ru_2_ pair (Ru5, Ru6) is more‐or‐less perpendicular to this plane (Figures 1c; Figure S3, Supporting Information). All Ru atoms are coordinated by three CO ligands. Furthermore, Ru1 and Ru2 are bridged by iodine with Ru–I distances of 268.2(1) and 268.7(1) pm (**Figure** **2**a). Whereas Ru1 and Ru2 have two Ru–Ru bonds, all other Ru atoms (Ru3 to Ru6) exhibit three Ru–Ru bonds. The Ru–Ru distances range from 295.0(1) (Ru3–Ru5) to 301.6(1) pm (Ru1–Ru4) (Figure 2b). These values are slightly elongated in comparison to [Ru_3_(CO)_12_] (283.7‐285.9 pm)^[^ ^11^ ^]^ or [Ru(CO)_4_]_n_ (286 pm),^[^ ^10^ ^]^ but they fit well with [Ru_3_(CO)_9_(µ_3_‐Ge^t^Bu)_2_] (295.4‐296.2 pm) (**Table** **1**).^[^ ^6c^ ^]^

**Figure 2:**
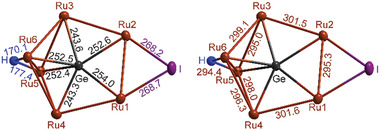
GeRu_6_ cluster core with selected distances (in pm), including H and I atoms (without CO ligands for clarity).

**Table 1:** Selected distances in [GeRu_6_(CO)_18_HI] in comparison to literature data.

Compound	Ge–Ru distance [pm]	Ru–Ru distance [pm]
[GeRu_6_(CO)_18_HI]	243.3(1)−243.6(1) 252.4(1)−254.0(1)	295.0(1)−301.6(1)
[Ru_3_(CO)_9_(µ^3^‐Ge*t*Bu)_2_] [6c]	245.3‐247.1	295.4‐296.2
[Ru_4_(CO)_10_(µ_4_‐Ge^t^Bu)_2_(µ‐Ge*^t^* BuH)_2_] [6c]	251.1‐261.2	283.1, 294.7
[Ru_5_(CO)_11_(µ‐GePh_2_)_4_(µ_5_‐C)] [6a]	247.3‐250.6	281.9‐289.7
[(µ_3_‐Ge{Ru(CO)_2_(µ_5_‐C_5_Me_4_H)})_2_Ru_3_(CO)_9_] [6b]	243.5‐254.9	288.6‐293.8
[Ru(CO)_4_]_n_ [10]	/	286.0
[Ru_3_(CO)_12_] [11]	/	283.7‐285.9

Two sets of Ru–Ge distances are observed for [GeRu_6_(CO)_18_HI] (Figure 2a). Shorter distances (243.3(1), 243.6(1) pm) occur to the in‐plane Ru atoms with three Ru neighbors (Ru3, Ru4), whereas longer distances (252.4(1)−254.0(1) pm) are observed to those Ru atoms above and below the plane (Ru5, Ru6) as well as to those Ru atoms bound to iodine (Ru1, Ru2). With 71.3(1)° (Ru5–Ge–Ru6) to 74.1(1) (Ru3–Ge–Ru6), all Ru–Ge–Ru angles are close to the ideal angle in a pentagon (72°). Furthermore, the Ru–C distances range from 188.5(5) (Ru5–C13) to 195.7(4) pm (Ru1–C3) and the C–O distances from 111.5(5) (C14–O14) to 114.8(5) pm (C11–O11). Both Ru–C and C–O distances are in good agreement with Ru_3_(CO)_12_ (Ru–C: 1.85‐1.93 pm; C–O: 1.13‐1.22 pm).^[^ ^11^ ^]^ Finally, the hydrogen atom bridging Ru5 and Ru6 is again located in the plane of the four Ru atoms and the I atom and exhibits Ru–H distances of 170 and 177 pm (Figure 2). Here, it should be noticed that the hydrogen atom could be refined without any constraints in the single‐crystal structure analysis.

To examine structure and bonding of the title compound, first of all, UV‐Vis and FT‐IR spectroscopy were performed. UV‐Vis indicates a strong absorption below 500 nm, which is in accordance with the red color of the title compound (Figure S5, Supporting Information). FT‐IR elucidates the situation from the perspective of the carbonyl ligands (Figure S6, Supporting Information). Here, characteristic CO vibrations are observed between 2115 and 1974 cm^–1^ with the strongest vibration at 2053 cm^–1^. This is in agreement with the carbonyl Ru_3_(CO)_12_ (2060‐2010 cm^–1^; strongest vibration at 2060 cm^–1^; Figure S6b, Supporting Information),^[^ ^12^ ^]^ which also indicates the valence state of Ru to be similar to Ru_3_(CO)_12_ – i.e., ±0. In contrast, the CO vibrations of *cis*‐Ru^+II^(CO)_4_I_2_ occur at significantly higher wavenumbers (2160‐2066 cm^–1^, **Table** **2**).^[^ ^12^ ^]^

**Table 2:** CO vibrations of [GeRu_6_(CO)_18_HI] in comparison to literature data (vs: very strong, s: strong, m: medium, w: weak, vw: very weak).

Compound	CO vibration/cm^–1^
[GeRu_6_(CO)_18_HI]	2125 (vw), 2094 (w), 2082 (w), 2053 (s), 2023 (w), 2013 (m), 1989 (w), 1974 (w)
[Ru_3_(CO)_12_] [12]	2060 (vs), 2029 (s), 2010 (m)
[*cis*‐RuI_2_(CO)_4_] [12]	2160 (m), 2119 (vw), 2105 (vs), 2095(s), 2066 (s)

Based on crystal structure analysis and FT‐IR spectroscopy, ruthenium can be considered to be zerovalent (Ru^±0^) with non‐charged CO ligands. In regard to their electronegativity, hydrogen and iodine can be assumed as negatively charged (H^–I^, I^–I^), which, in turn, leads to divalent germanium (Ge^+II^). In regard to the 18‐electron‐rule, which is often relevant for carbonyl clusters, the cluster compound is obviously electron deficient with 108 e^–^ (6×18 e^–^) expected and 92 e^–^ available (Ru^±0^: 6×8 e^–^; CO ligands: 2 × 3 × 6 e^–^; Ge^+II^: 2 e^–^; H^–I^: 2e^–^; I^–I^: 4 e^–^). Moreover, the planar GeRu_4_HI unit is surprising and different from the most often observed closed‐shell arrangements of carbonyl cluster compounds.^[^ ^6^, ^7^, ^13^ ^]^

In regard to structure and bonding, furthermore, electron paramagnetic resonance (EPR) spectroscopy, nuclear magnetic resonance (NMR) spectroscopy, mass spectrometry (MS), and density functional theory (DFT) calculations were performed. In this regard, ESR shows the absence of unpaired electron spins in [GeRu_6_(CO)_18_HI] and, thus, indicates diamagnetic behavior (Figure S7, Supporting Information). NMR spectroscopy (^1^H, ^99^Ru, ^73^Ge) unfortunately was not indicative due to the insufficient solubility of [GeRu_6_(CO)_18_HI] (≤2 mg mL^−1^ in THF‐d8/CDCl_3_, required: >5 mg mL^−1^ for ^1^H‐NMR, >10 mg mL^−1^ for ^99^Ru/^73^Ge‐NMR; Table S2, Figure S8, Supporting Information). According to high‐resolution MS isotopic patterns, its exact mass and the most intense peak (1112.349 m/z) point to [Ru_6_C_18_O_18_H]^–^ (**Figure** **3**a,b), which is in agreement with the title compound after the detachment of GeI^+^. The exact mass of the cluster unequivocally confirms the presence of the hydrogen atom. The stability of a hydrogen atom connected to a ruthenium carbonyl cluster anion is to be expected and was reported before in MS‐monitored gas‐phase processes.^[^ ^14^ ^]^ Besides crystal structure analysis (H atom refined without constraints) and MS, the presence of hydrogen is also confirmed by FT‐IR spectroscopy (Figure 3c; Figure S9, Supporting Information). In this regard, density functional theory (DFT) calculations (B3LYP‐D3(BJ) level) allowed to identify three IR‐active vibrations with hydrogen involved (Tables S3,S4, Figure S10, Supporting Information): 1387 cm^–1^ (*ν_asym_* (Ru–H), B_1_), 1277 cm^–1^ (*ν_sym_* (Ru–H), A_1_), 718 cm^–1^ (*δ*(Ru_2_H), B_2_) with an expected intensity B_2_ > B_1_ > A_1_. While the range 1350–1250 cm^–1^ is superimposed by the ionic liquid, weak vibrations are indeed observed at 1399 and 723 cm^–1^ (indicated by arrows: Figure 3c).

**Figure 3:**
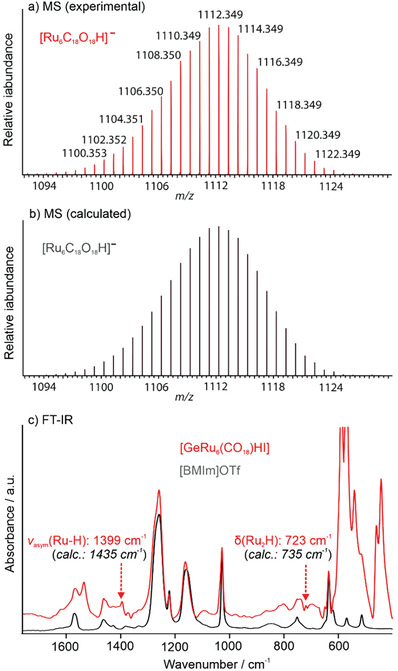
MS (a,b) of [GeRu_6_(CO)_18_HI] with experimental a) and calculated data b) of a [Ru_6_C_18_O_18_H]^–^ fragment as well as FT‐IR spectrum of [GeRu_6_(CO)_18_HI] c) with Ru–H vibrations indicated by arrows (grey: [BMIm][OTf] as a reference).

Finally, DFT calculations (see Supporting Information) were used to elucidate structure and bonding in [GeRu_6_(CO)_18_HI] (Figure 4; Tables S5–S7, Supporting Information). Hence, the bonding situation can be rationalized based on seven Ru–Ge–Ru two‐electron‐three‐center (2e3c) bonds, two Ru–I two‐electron‐two‐center (2e2c) bonds, and one Ru–H–Ru 2e3c bond (**Figure** **4**b–g). The 18‐electron rule is obeyed for each of the six Ru atoms. Together with 84 e^–^ from Ru^±0^ and the CO ligands, the 2 e^–^ from Ge^+II^, the 2 e^–^ from H^−^, and the 4 e^–^ from the two 2e2c bonds to I^−^ yield a total of 92 e^–^. Furthermore, all eight 2e3c bonds are shared by two Ru centers, must hence be double counted, and add another 16 e^–^ to finally yield 108 e^–^ as requested by the 18‐electron rule. From the viewpoint of the Ru1 or Ru2 atom, the three Ru–CO bonds contribute 12 e^–^ and another 6 e^–^ reside in one 2e2c bond and two 2e3c bonds about the atom. The atoms Ru3 through Ru6 have three CO ligands (12 e^–^) and three 2e3c bonds (6 e^–^) about the atom, and thus also are surrounded by 18 valence electrons.

**Figure 4:**
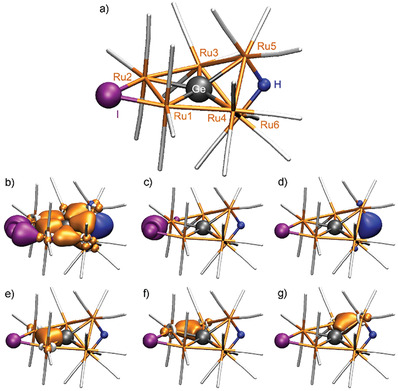
DFT calculation of [Ru_6_GeHI(CO)_18_]: a) Optimized equilibrium geometry of the [Ru_6_GeHI(CO)_18_] cluster as obtained at the PBE0‐D4/def2‐TZVP level; b–g) Localized molecular orbitals obtained from a Boys localization procedure applied to the PBE0‐D4/def2‐TZVP canonical orbitals shown for an isovalue of $\pm 0.1\;a_{0}^{-3/2}$ with b) two Ru–I two‐electron‐two‐center (2e2c) bonds, seven Ru–Ge–Ru two‐electron‐three‐center (2e3c) bonds, and one Ru–H–Ru 2e3c bond; c) one of the two Ru–I 2e2c bonds; d) Ru–H–Ru 2e3c bond; e‐g) three non‐equivalent Ru–Ge–Ru 2e3c bonds.

The integration of magnetically induced current densities yields a substantial σ‐type diatropic net‐ring current of about 10 nA T^−1^ with the planar GeRu_4_HI unit being involved (**Figure** **5**; Figure S11, Supporting Information). On the right‐hand side, it is split into two parts (Ru3–Ru5–Ru4 and Ru3–Ru6–Ru4), each amounting to about 3.5 nA T^−1^. The remainder flows across the H atom. In fact, the net‐ring current of GeRu_4_HI is similar to the π‐type net‐ring current of benzene (11.9 nA T^−1^).^[^ ^15^ ^]^ The special bonding situation in [GeRu_6_(CO)_18_HI] with the Ru–Ge–Ru 2e3c bonds and σ‐aromaticity are also in close relation with the flat appearance of [GeRu_6_(CO)_18_HI] and the planar GeRu_4_HI unit.

**Figure 5:**
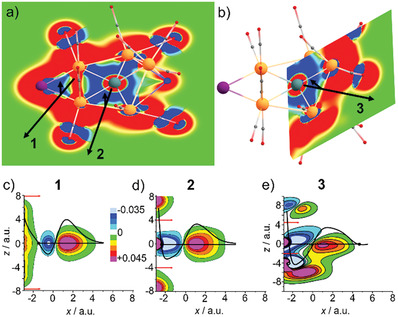
Magnetically induced current densities *j* for a B field perpendicular to the plane I–Ru3–Ru4. Upper row: absolute value of *j* in the planes I–Ru3–Ru4 a) and Ge‐Ru4‐Ru5 b). Red/blue colors indicate dia‐/paratropic currents greater than 0.04 a.u. The black arrows show the positions of the planes in c) to e). Lower row: contour diagrams of the component of *j* perpendicular to the planes defined in (a) and (b). The red arrows mark the upper and lower boundaries for the integration in the z direction to obtain the current profiles (black curves, arbitrary units). Additional integration in the x direction for the boundaries indicated by the points in the current profile yields diatopic net currents amounting to 11.5 nA/T c), 9.9 nA/T d), and 3.5 nA/T e).

In summary, ionic‐liquid‐based synthesis results in the novel carbonyl cluster compound [GeRu_6_(CO)_18_HI], which shows unique structural features and an unusual, complex bonding situation. On the one hand, this comprises a Ge‐centered five‐membered Ru_4_(Ru_2_) ring as the central open‐shell cluster core. Moreover, an unexpected planar GeRu_4_HI unit is observed. The molecular compound is non‐charged and features only paired electron spins. The oxidation states can be concluded to be Ru^±0^, Ge^+II^, H^–I^, and I^–I^. The bonding situation can be rationalized based on extensive two‐electron‐three‐center bonding (7× Ru–Ge–Ru, 2× Ru–H–Ru), which results in the 18‐electron rule being obeyed for all Ru atoms. The special bonding situation, also including σ‐aromaticity with a substantial ring current of 9–10 nA T^−1^, finally, explains the planar GeRu_4_HI unit and the overall structure of the [GeRu_6_(CO)_18_HI] carbonyl cluster compound. In summary, [GeRu_6_(CO)_18_HI] is a unique example of a non‐carbon compound with substantial aromaticity and a complex bonding situation, which illustrates what is possible in fundamental chemistry with elements and bonding.

Supporting Information: Details related to analytical techniques, synthesis, structural characterization, spectroscopic characterization, computation. This material is available from the Wiley Online Library. Further details of the crystal structure analysis may be also obtained from the joint CCDC/FIZ Karlsruhe deposition service by quoting the depository number 2 260 888.

## Conflict of Interest

The authors declare no conflict of interest.

## Supporting information

Supporting Information

Supporting Information

Supporting Information

## Data Availability

The data that support the findings of this study are available from the corresponding author upon reasonable request.
