# Supplementary material for: [GeRu6(CO)18HI]: A Germanium‐Centered Ruthenium Carbonyl Cluster with Aromatic Ring Current
Source: Adv Sci (Weinh). 2024 Mar 21;11(21):2309043. doi: 10.1002/advs.202309043 (PMC11151064; doi:10.1002/advs.202309043)
Supplement: Supplementary file 1 — Supporting Information [file ADVS-11-2309043-s003.pdf]

ADVANCED SCIENCE
Open Access

Supporting Information

for *Adv. Sci.*, DOI 10.1002/advs.202309043

[$GeRu_6(CO)_{18}HI$]: A Germanium-Centered Ruthenium Carbonyl Cluster with Aromatic Ring Current

*Silke Wolf, Ralf Köppe, Jens Treptow, Wolfram Feuerstein, Jonas Wenzel, Frank Breher, Peter W. Roesky, Florian Weigend, Wim Klopper and Claus Feldmann**

[$GeRu_6(CO)_{18}HI$]: A Germanium-Centered Ruthenium Carbonyl Cluster with Aromatic Ring Current

Silke Wolf[a], Ralf Köppe[a], Jens Treptow[a], Wolfram Feuerstein[a], Jonas Wenzel[a], Frank Breher[a], Peter W. Roesky[a], Florian Weigend[b], Wim Klopper[c], and Claus Feldmann[a]*

–SUPPORTING INFORMATION–

[a] Dr. S. Wolf, Dr. R. Köppe, J. Treptow, Dr. W. Feuerstein, MSc. J. Wenzel, Prof. Dr. F. Breher, Prof. Dr. P. W. Roesky, Prof. Dr. C. Feldmann
Institute for Inorganic Chemistry
Karlsruhe Institute of Technology (KIT)
Engesserstraße 15, D-76131 Karlsruhe (Germany)
E-mail: claus.feldmann@kit.edu

[b] Prof. Dr. F. Weigend
Fachbereich Chemie
Philipps-Universität Marburg
Hans-Meerwein-Strasse 4, D-35043 Marburg (Germany)

[c] Prof. Dr. W. Klopper
Institute of Physical Chemistry
Karlsruhe Institute of Technology (KIT)
Fritz-Haber-Weg 2, D-76131 Karlsruhe (Germany)

Contents

1. Analytical Techniques

2. Synthesis

3. Structural Characterization

4. Spectroscopic Characterization

5. Computation

1. Analytical Techniques

Single-crystal X-ray structure analysis. For single crystal structure analysis, suitable crystals of [$GeRu_6(CO)_{18}HI$] were selected, covered by inert-oil (perfluoropolyalkylether, ABCR, Germany), and placed on a micro gripper (MiTeGen, USA). Data collection was performed at 200 K on an IPDS II image-plate diffractometer (Stoe, Germany) using Mo-K_α radiation (λ = 0.71073 Å, graphite monochromator) as well as at 180 K on a Stoe StadiVari Diffractometer with Euler geometry (Stoe) using Ga–K_α radiation (λ = 1.34013 Å, graded multi-layer mirror as monochromator). Data reduction and multi-scan absorption correction were conducted by the X-AREA software package (version 1.75) and STOE LANA (version 1.75).[S1] Space group determination based on systematic absence of reflections was performed by XPREP.[S2] Using Olex2[S3], the structure was solved with the ShelXT[S4] structure solution program using Intrinsic Phasing and refined with the ShelXL[S4] refinement package using least squares minimization. All non-hydrogen atoms were refined anisotropically. The bridging H-atom was located directly from the maximum residual density, assigned a fixed thermal displacement parameter and its coordinates were refined freely. Detailed information on crystal data and structure refinement are listed in Table S1. DIAMOND was used for all illustrations[S5] Further details of the crystal structure investigation may be obtained from the joint CCDC/FIZ Karlsruhe deposition service on quoting the depository number CSD-No. 2260888.

Energy dispersive X-ray spectroscopy (EDXS) was performed using an Ametec EDAX mounted on a Zeiss SEM Supra 35 VP scanning electron microscope (Zeiss, Germany). The samples were prepared in the glove-box by selecting single crystals that were fixed on a conductive carbon pad on an aluminum sample holder. The samples were handled under inert conditions during transport and sample preparation.

Fourier-transformed infrared (FT-IR) spectra were recorded on a Bruker Vertex 70 FT-IR spectrometer (Bruker, Germany). The samples were measured as pellets in KBr. Thus, 300 mg of dried KBr and 0.5-1.0 mg of the sample were carefully pestled together and pressed to a thin pellet.

Optical spectroscopy (UV-Vis) of powder samples was recorded on a Shimadzu UV-2700 spectrometer (Shimadzu, Japan), equipped with an integrating sphere, in a wavelength interval of 250-800 nm against $BaSO_4$ as reference.

Continuous-wave electron paramagnetic resonance (CW-EPR) spectroscopy. CW-EPR measurements (X-band) were performed with a Bruker EMXplus spectrometer (Bruker, Germany). Field calibration was performed using 2,2-diphenyl-1-picrylhydrazine (DDPH) with a g-value of 2.0036 (solution in toluene; MF: 9.424060 GHz; center frequency: 3500 G; sweep width: 500 G; modulation amplitude: 4.42 G; modulation frequency: 100.00 kHz; time constant: 40.96 ms; conversion time: 160.00 ms; number of scans: 10; resolution in X: 1024).

Nuclear magnetic resonance (NMR) spectroscopy. ^{1}H-NMR spectroscopy was performed on a Bruker Avance II, operating at 300 MHz (Bruker, Germany). For this purpose, a saturated solution of [$GeRu_6(CO)_{18}HI$] in $CDCl_3$ (2 mg/mL; *compare Table S2*). The resulting solution was analyzed directly after preparation. Chemical shifts were referenced internally using signals of the residual protio solvent (^{1}H) were reported relative to tetramethylsilane. All NMR spectra were measured at 298 K, unless otherwise specified.

Mass spectrometry (MS). Electro spray ionization (ESI) mass spectra were recorded on a Q Exactive (Orbitrap) mass spectrometer (Thermo Fisher Scientific, San Jose, CA, USA) equipped with a HESI II probe at a capillary temperature of 320 °C. The FT resolution was set to 140,000. The instrument was calibrated in the m/z range 128-1800 with a mass deviation of less than 1 ppm. All spectra were recorded in the negative mode. The sample was prepared in d^8 toluene as solvent in a glove box under argon at a concentration of about 0.1 mg/mL. The flow rate was set to 5 µL/min.

2. Synthesis

General. All reactions and sample handling were performed with dried argon atmosphere using standard Schlenk techniques or glove boxes. Reactions were performed in Schlenk flasks and glass ampoules that were evacuated ($p < 10^{-3}$ mbar), heated and flashed with argon three times prior to use. The starting materials GeI_4 (99.99%, ABCR, Germany) and $Ru_3(CO)_{12}$ (99%, ABCR) were used as received. [BMIm][OTf] (high purity, IoLiTec, Germany) was dried under reduced pressure (10^{-3} mbar) at 100 °C for 48 h. All compounds were handled and stored in argon-filled glove boxes (c(O_2, H_2O) < 0.1 ppm, MBraun Unilab; MBraun, Germany).

[GeRu$_6$(CO)$_{18}$HI]. 80 mg (0.1379 mmol) of GeI_4, 88.2 mg (0.1379 mmol) of $Ru_3(CO)_{12}$ and 1 mL of [BMIm][OTf] were heated under argon in a sealed glass ampoule for 96 h at 130 °C. After cooling to room temperature with a rate of 1 K/h, the title compound crystallizes as orange to red crystals and was obtained as a side phase (about 20% yield) together with a black powder of $[Ru(CO)_4]_n$ (Figure S1). The title compound is highly sensitive to air and moisture and needs to be handled with strict inert conditions. Since $[Ru_6GeHI(CO)_{18}]$ could not be obtained as a pure phase, crystals for characterization were manually separated from the side phase.

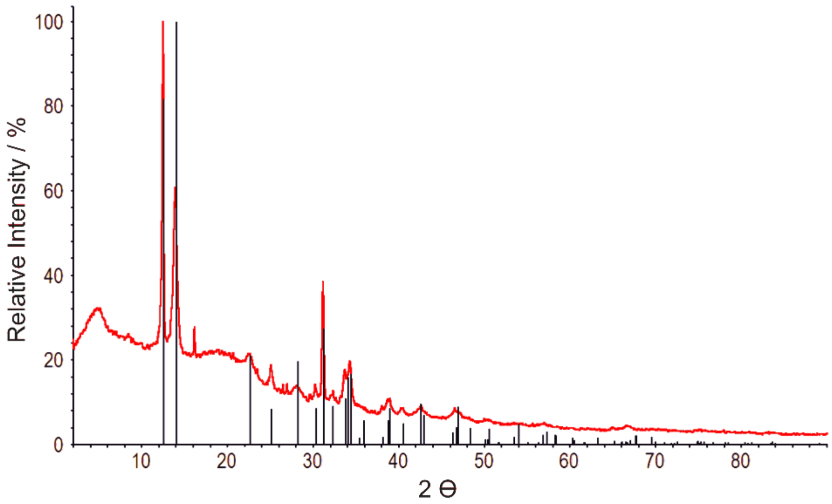

Figure S1. XRD indicating $[Ru(CO)_4]_n$ as a side product of the synthesis of $[GeRu_6(CO)_{18}HI]$ (ICDD-No. 01-081-1204: $[Ru(CO)_4]_n$ as a reference).

3. Structural Characterization

Structural data and refinement details are summarized in Table S1. The unit cell of the title compound is displayed in Figure S2. Moreover, the molecular structure of [$GeRu_6(CO)_{18}HI$] is shown with different views (Figure S3). Finally, the long-ranging distances between the molecular units of [$GeRu_6(CO)_{18}HI$] are displayed in Figure S4. Accordingly, the shortest intermolecular H–I distances are 654.3(7) and 669.7(7) pm (Figure S4a,b). The shortest intermolecular Ru–Ru distances are 608.7(1) and 613.4(1) pm (Figure S4c).

Table S1. Crystallographic data and refinement details of [$GeRu_6(CO)_{18}HI$].

**Data**	**[$GeRu_6(CO)_{18}HI$]**
Sum formula	$C_{18}HO_{18}GeIRu_6$
Formula weight	1311.1 g mol^{-1}
Crystal system	monoclinic
Space group	$P2_1/c$
Lattice parameters	a = 892.8(1) pm<br>b = 1659.1(1) pm<br>c = 2243.3(6) pm<br>β= 112.7(1) °
Cell volume	V = 3066.0×10^6 pm^3
Formula units per cell	Z = 4
Calculated density	ρ = 2.84 g cm^{-3}
Measurement limits	–11 ≤ h ≤ 8, –21 ≤ k ≤ 21, –16 ≤ l ≤ 29
Theta range for data collection	8.8 to 125.1°
Measurement conditions	Stoe StadiVari<br>λ(Ga–Kα) = 134.143 pm<br>T = 180(2) K
Linear absorption coefficient	μ = 22.46 mm^{-1}
Number of reflections	17896 (7046 independent)
Refinement method	Full-matrix least-squares on F^2
Merging	R_{int} = 0.021
Number of parameters	401
Residual electron density	1.11 to –0.99 e$^-$·10^{-6} pm^{-3}
Figures of merit	R1 (I ≥ 2σ_I) = 0.025<br>R1 (all data) = 0.029<br>wR2 (all data) = 0.069<br>GooF = 1.06

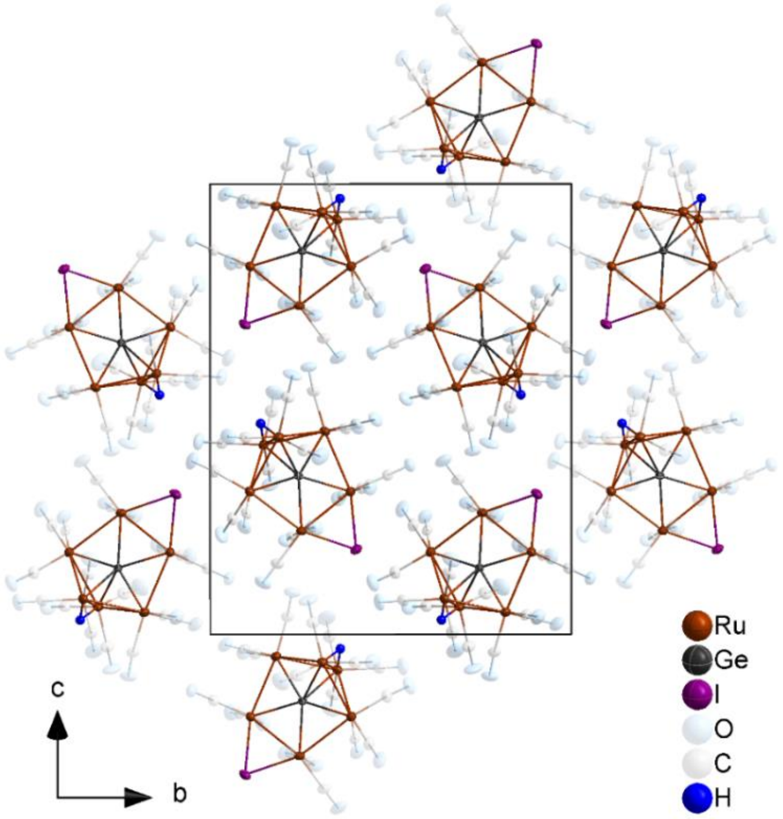

Figure S2. Unit cell of [$Ru_6GeHI(CO)_{18}$].

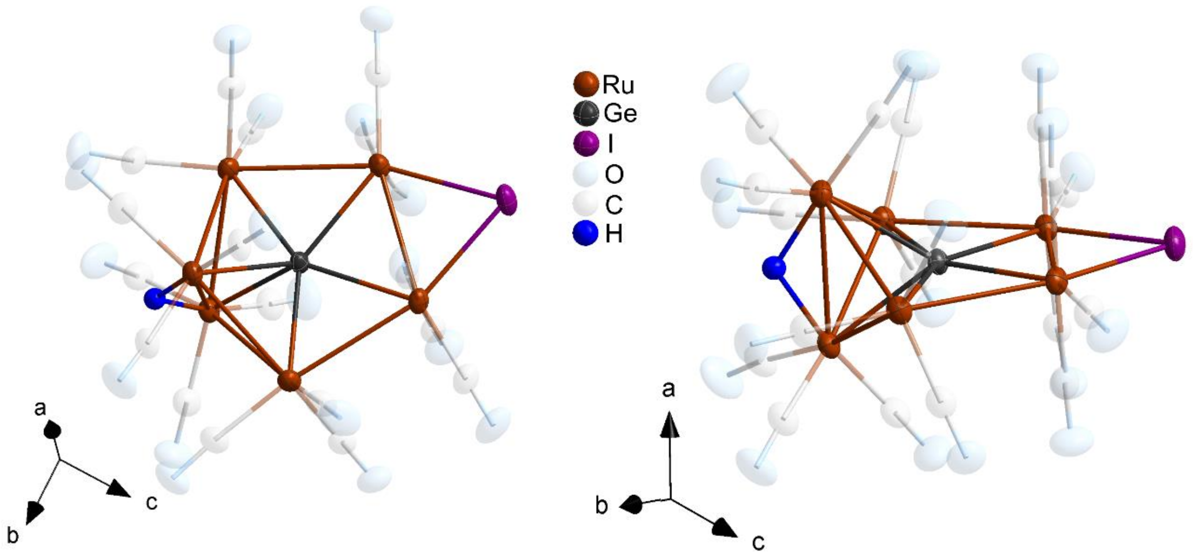

Figure S3. Molecular structure of [$GeRu_6(CO)_{18}HI$] with top view and side view.

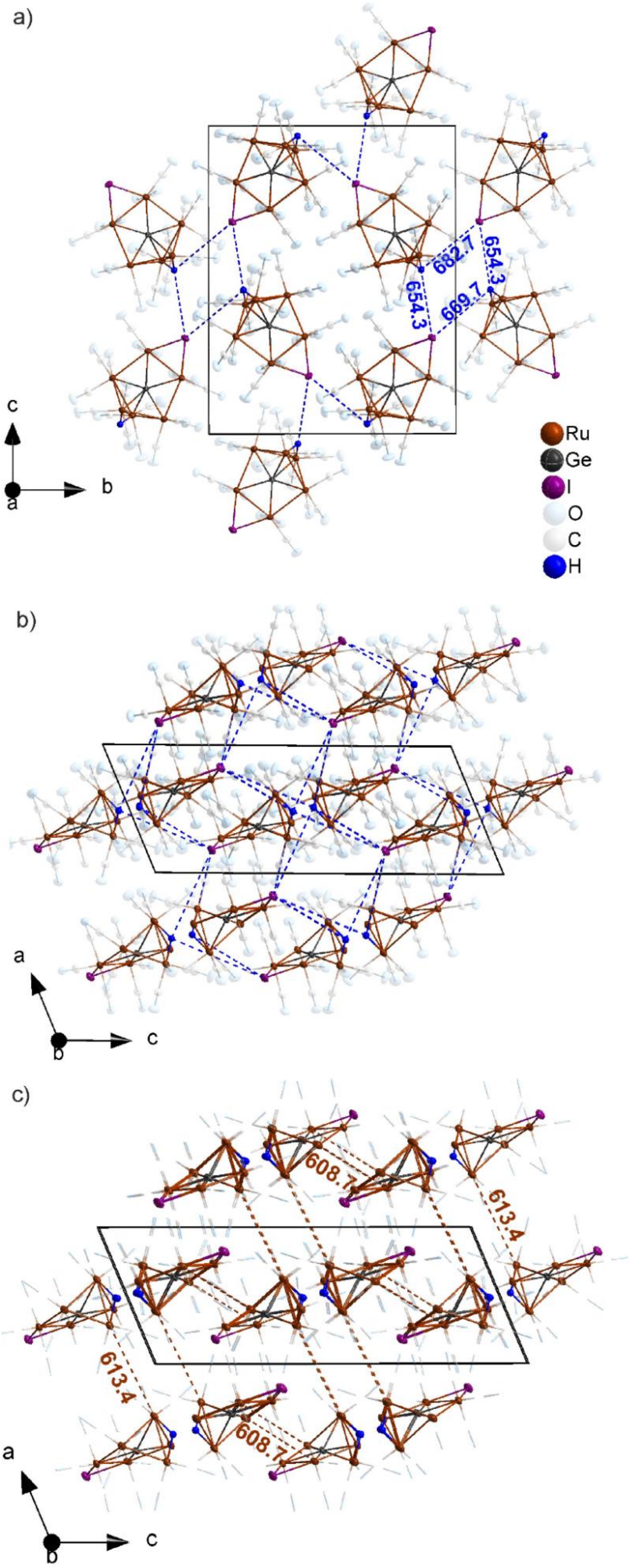

Figure S4. Intramolecular distances between [$GeRu_6(CO)_{18}HI$]: a+b) shortest H–I distances (blue), c) shortest Ru–Ru distances (brown) (distances in pm).

4. Spectroscopic Characterization

To verify the red color of the title compound, UV-Vis spectroscopy was performed (Figure S5). Here strong absorption is observed below 500 nm, which is in accordance with the red color of single crystals (*see main paper: Figure 1b*).

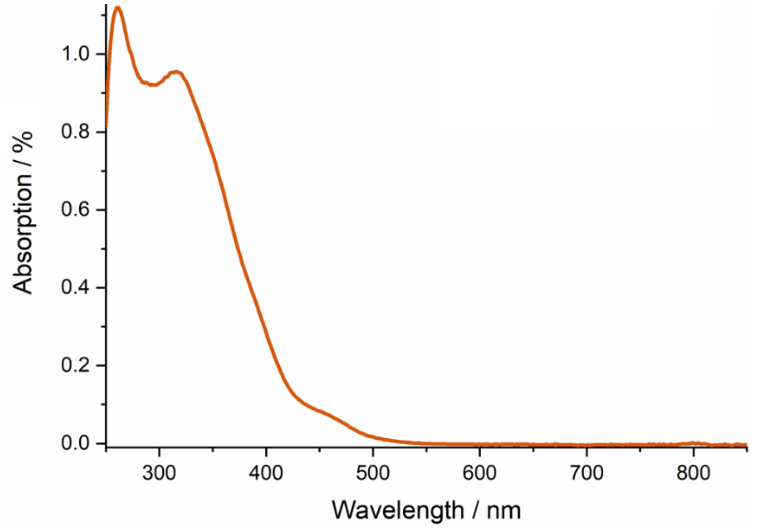

Figure S5. UV-Vis spectrum of [$GeRu_6(CO)_{18}HI$].

FT-IR elucidates the situation from the perspective of the carbonyl ligands (*SI: Figure S6*). Characteristic CO vibrations are observed between 2115 and 1974 cm^{-1} with the strongest vibration at 2053 cm^{-1}. Beside the vibrations of [$GeRu_6(CO)_{18}HI$], additional vibrations originate from the ionic liquid remaining adhered on the crystal surfaces.

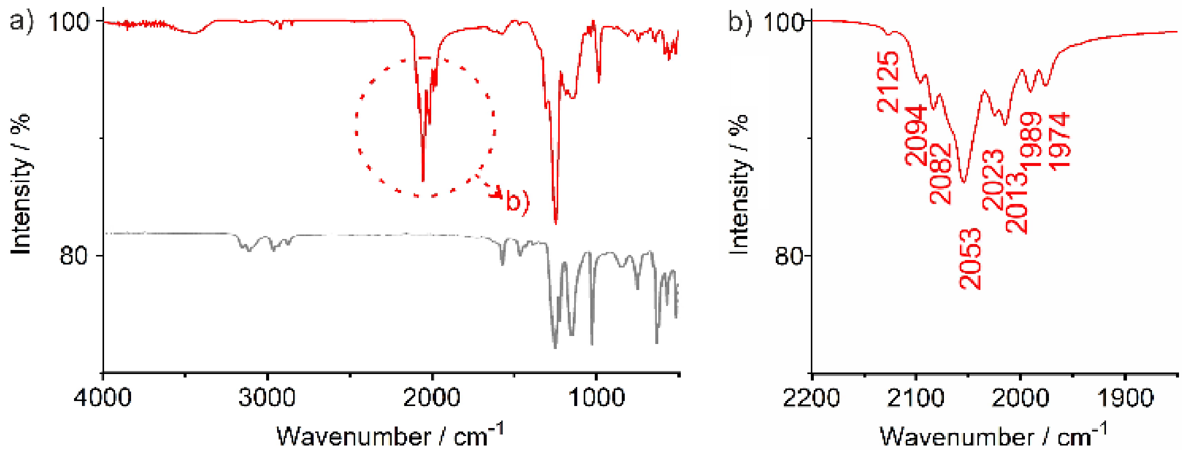

Figure S6. a) FT-IR spectra of [$GeRu_6(CO)_{18}HI$] with b) detailed view of the CO vibrations (grey: [BMIm][OTf] as a reference).

Continuous-wave electron paramagnetic resonance (CW-EPR) spectroscopy was performed in toluene. Here, no specific signal could be detected (Figure S7). Accordingly, [$GeRu_6(CO)_{18}HI$] does not exhibit any electron spin due to unpaired

electrons. As a result, [$GeRu_6(CO)_{18}HI$] is diamagnetic. Here, it needs to be noticed that electron spins were only detected after beginning decomposition of the title compound and originate from decomposition products.

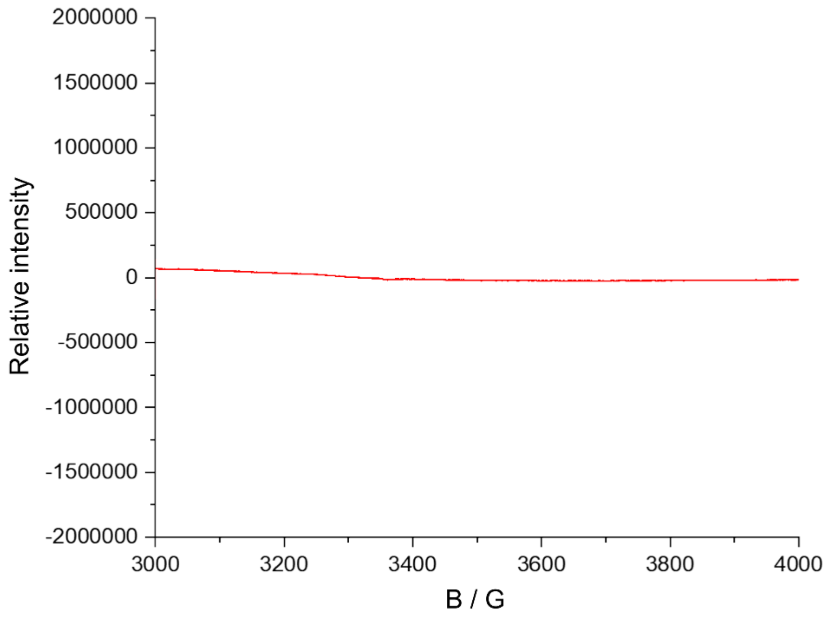

Figure S7. CW-EPR spectrum of [$GeRu_6(CO)_{18}HI$] (solution in toluene).

Nuclear magnetic resonance spectroscopy (^{1}H-NMR) was performed to prove the presence of the hydrogen atom in [$GeRu_6(CO)_{18}HI$]. To this concern, the solubility of the title compound was evaluated in different solvents (Table S2) at different temperatures (–80 to +80 °C). The dissolution of [$GeRu_6(CO)_{18}HI$] is visually indicated by the orange to yellow color of the solvent (Figure S8b). However, the solubility is limited to about 2 mg/mL in THF or $CHCl_3$, and about 1 mg/mL in toluene at room temperature (Table S2). In DMSO, the title compound decomposes instantaneously with formation of a grey precipitate (Ru↓) and gas evolution (CO↑). In hexane, [$GeRu_6(CO)_{18}HI$] is insoluble. At room temperature, the title compound decomposes slowly over a period of some days even in THF, $CHCl_3$ and toluene as indicated by the color change of the solution from orange to yellow to colorless (Table S2). The decomposition is slower at lower temperatures (–80 °C) but the solubility is as well significantly decreased at low temperature.

Based on the aforementioned limitations regarding solubility, chemical and thermal stability, ^{1}H-NMR spectra were recorded with saturated solutions of [$GeRu_6(CO)_{18}HI$] in $CDCl_3$ at –80°C. The presence of a saturated solution was indicated by the presence of a solid precipitate remaining at the bottom of the solution (Figure S8b). Based on these conditions, no ^{1}H signal for [$GeRu_6(CO)_{18}HI$] was observed (Figure S8a). Signals at +5 to 9 ppm originate from traces of the ionic liquid ([BMIm][OTf]) that remained absorbed on the crystal surfaces of [$GeRu_6(CO)_{18}HI$]. According to computed ^{1}H chemical shifts (*see SI: chapter 5*), the ^{1}H signal for [$GeRu_6(CO)_{18}HI$] would have to be expected at –15 and –25 ppm (*compare Table S5*). In sum, the absence of any ^{1}H signal can be atributed to the low solubility of the title compound. Thus, ^{1}H-NMR would require > 5 mg/mL. For ^{99}Ru or ^{73}Ge-NMR, the required amounts in solution can be estimated to be even higher (>10 mg/mL).

Table S2. Solubility of [$GeRu_6(CO)_{18}HI$] in different solvents at room temperature.

Solvent	Solubility	Experimental observation
**DMSO**	Decomposition	Color change from orange solution to colorless solution with grey precipitate and gas evolution
**THF**	2 mg/mL	Stable for 1-2 days; thereafter slow color change from orange solution via yellow to colorless solution
**$CHCl_3$**	2 mg/mL	Stable for 1-2 days; thereafter slow color change from orange solution via yellow to colorless solution
**Toluene**	1 mg/mL	Stable for 1-2 weeks; thereafter slow color change from orange solution via yellow to colorless solution
**Hexane**	Insoluble	/

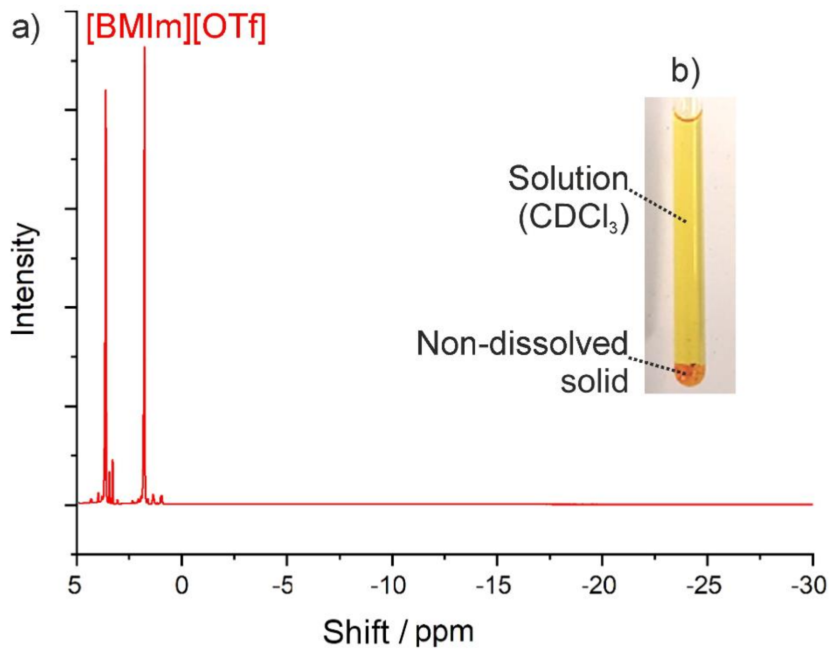

Figure S8. ^1H-NMR spectrum of [$GeRu_6(CO)_{18}HI$] (–80°C, solution in $CDCl_3$) (signals at 5 ppm to 0 ppm originate from residual [BMIm][OTf] as ionic liquid).

In addition to the large-scale FT-IR spectrum (1300-600 cm^{-1}) shown in the main manuscript (*Figure 3c*), the full spectrum is shown here in Figure S9.

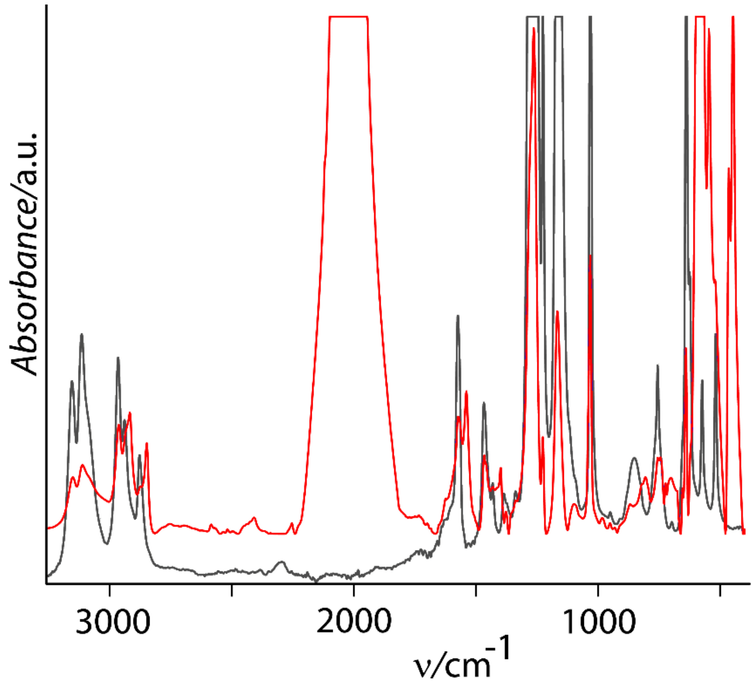

Figure S9. FT-IR spectrum (red) of [$GeRu_6(CO)_{18}HI$] (black: [BMIm][OTf] as a reference).

5. Computation

Quantum-chemical computations were performed at the level of density-functional theory (DFT) with the TURBOMOLE program package.[S6] The dispersion corrections D4[S7] and D3 were applied, the latter with the Becke-Johnson damping function, that is, D3(BJ).[S8] The functionals TPSS,[S9] TPSSh,[S10] PBE,[S11] PBE0,[S12] ωB97X,[S13] and B3LYP[S14] were employed. All computations with these functionals were carried out in the def2-TZVP basis set.[S15] TURBOMOLE's quadrature grid 5 was used and weight derivatives were considered when computing the analytic nuclear gradients. The geometry optimizations were performed in C_{2v} symmetry, the self-consistent-field convergence threshold (scfconv) was set to $10^{-10}\ E_\mathrm{h}$, and an equilibrium geometry was considered to be converged when the energy was converged to within $10^{-9}\ E_\mathrm{h}$ and the norm of the Cartesian gradient vector was less than $10^{-6}\ E_\mathrm{h}/a_0$. All equilibrium geometries were confirmed to be minima on the potential-energy hypersurface by computing (with TURBOMOLE's AOFORCE module) the harmonic vibrational frequencies, which were all real.

Harmonic vibrational frequencies of the three vibrations that involve the H atom are given in Table S3. Anharmonic corrections were obtained by computing potential energy curves along the three normal modes that involve the H atom at the PBE0-D4 and B3LYP-D3(BJ) levels (Table S4). To illustrate the procedure, three potential energy curves were obtained from PBE0-D4 calculations (Figure S10).

Interatomic distances of the equilibrium geometries of [$GeRu_6(CO)_{18}HI$] using various functionals are listed in Table S5. Wiberg bond indices obtained in the def2-TZVP basis are listed in Table S6. Computed ^{1}H chemical shift (in ppm) for [$GeRu_6(CO)_{18}HI$] were calculated relative to tetramethylsilane and are listed in Table S7.

Magnetically induced current densities were calculated with GIMIC[S16] from the magnetic response density obtained with TURBOMOLE's module[S17] for chemical shielding constants at level PBE0/def2-TZVP. The integration boundaries for the currents were obtained from the zero-crossings of the current profiles; the outer boundaries for plane 1 and plane 2 were set to 8 a.u. (*see main paper: Figure 5*). Furthermore, contour plots of the σ-aromatic system are shown in Figure S11.

Table S3. Computed harmonic vibrational frequencies (ω_e in cm^{-1}) of vibrations involving the H atom of the [$GeRu_6(CO)_{18}HI$] cluster in C_{2v} symmetry. Also given is the computed IR intensity in km/mol. Four Ru atoms lie in the *y,z*-plane, two Ru atoms lie in the *x,z*-plane. The def2-TZVP basis was used.

	**$\delta(Ru_2H)$ (B_2)**		**ν_{sym}(Ru–H) (A_1)**		**ν_{asym}(Ru–H) (B_1)**	
	ω_e (cm^{-1})	Intensity (km/mol)	ω_e (cm^{-1})	Intensity (km/mol)	ω_e (cm^{-1})	Intensity (km/mol)
**TPSS-D3(BJ)**	683	37.57	1314	5.51	1398	0.78
**TPSS-D4**	692	2.00	1311	5.55	1399	0.57
**TPSSh-D3(BJ)**	701	39.71	1327	5.48	1421	0.01
**TPSSh-D4**	753	10.53	1322	5.57	1423	0.08
**PBE-D3(BJ)**	652	44.65	1274	7.10	1326	1.54
**PBE-D4**	653	44.07	1275	7.06	1325	1.54
**PBE0-D3(BJ)**	704	45.93	1320	6.64	1399	1.16
**PBE0-D4**	707	14.32	1317	6.70	1400	1.47
**B3LYP-D3(BJ)**	718	35.66	1277	6.26	1387	8.71
**B3LYP-D4**	717	35.65	1275	6.25	1386	9.34
**ωB97X-D3(BJ)**	757	43.46	1362	6.13	1437	7.99
**ωB97X-D4**	743	42.70	1297	6.30	1395	29.95

Table S4. Anharmonic corrections to the vibrational frequencies as computed at the PBE0-D4 and B3LYP-D3(BJ) levels. The def2-TZVP basis was used.

	**$\delta(Ru_2H)$ (B_2)**		**ν_{sym}(Ru–H) (A_1)**		**ν_{asym}(Ru–H) (B_1)**	
	ω_e (cm^{-1})	$\nu_{0\to1}$ (cm^{-1})	ω_e (cm^{-1})	$\nu_{0\to1}$ (cm^{-1})	ω_e (cm^{-1})	$\nu_{0\to1}$ (cm^{-1})
**PBE0-D4**	707	735	1317	1284	1400	1435
**B3LYP-D3(BJ)**	718	741	1277	1237	1387	1426

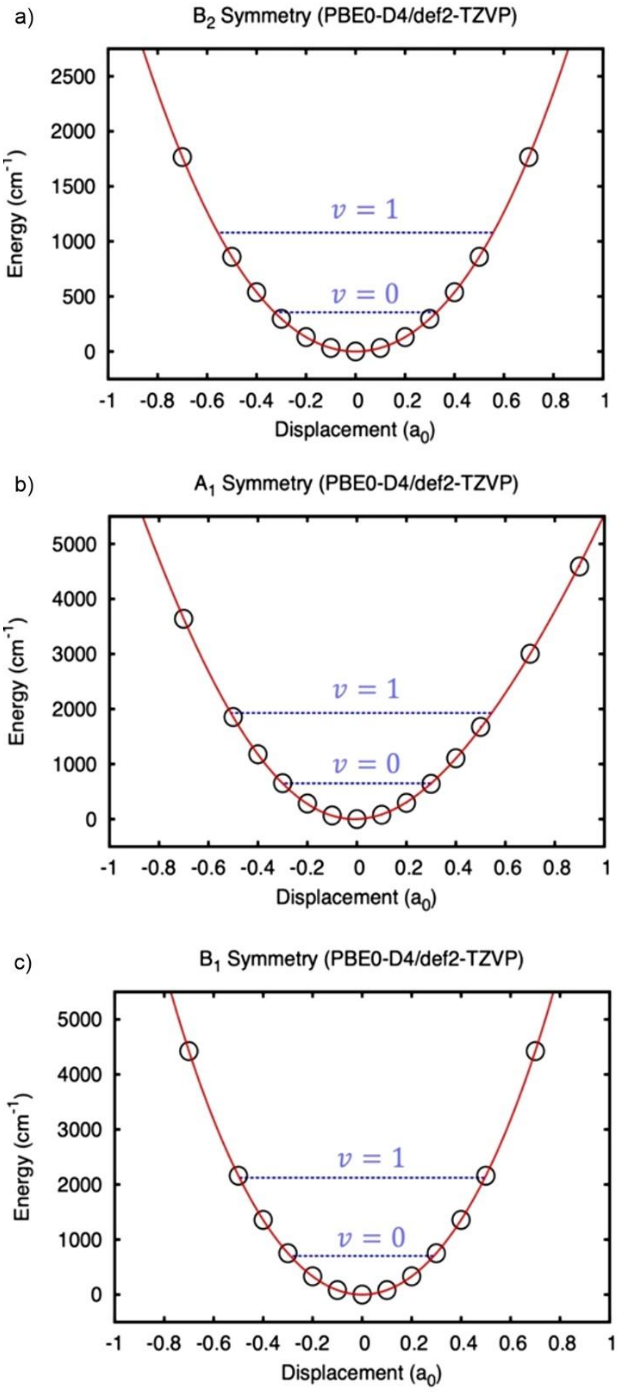

Figure S10. Potential energy curves computed along the normal modes a) δ (Ru_2H) (*B2*), b) ν_{sym} (Ru–H) (*A1*) and c) ν_{asym} (Ru–H) (*B1*) together with vibrational levels $v = 0$ and $v = 1$.

Table S5. Interatomic distances (in pm) of the equilibrium geometries of the [$GeRu_6(CO)_{18}HI$] cluster, optimized in the def2-TZVP basis set at the DFT level using various functionals.

	Ru1–I	Ru5–H	Ge–Ru1	Ge–Ru3	Ge–Ru5	Ru1–Ru2	Ru2–Ru3	Ru3–Ru5	Ru5–Ru6
**TPSS-D3(BJ)**	271.1	181.8	253.2	244.7	252.7	296.6	301.7	297.8	294.8
**TPSS-D4**	270.9	181.8	253.9	245.5	253.2	295.8	302.6	299.2	295.2
**TPSSh-D3(BJ)**	270.0	181.3	252.7	243.7	252.0	295.4	300.7	297.1	294.5
**TPSSh-D4**	269.7	181.3	253.8	245.0	252.9	294.4	302.1	299.3	295.2
**PBE-D3(BJ)**	271.5	182.5	256.1	247.4	254.7	299.8	304.3	301.2	296.5
**PBE-D4**	271.4	182.5	255.9	247.2	254.6	299.7	304.2	301.0	296.3
**PBE0-D3(BJ)**	268.4	181.1	253.6	244.0	252.3	295.9	300.8	298.2	295.0
**PBE0-D4**	268.3	181.1	254.4	244.8	252.9	295.4	301.8	299.6	295.5
**B3LYP-D3(BJ)**	273.3	181.6	256.7	246.1	254.1	299.0	303.2	301.6	300.1
**B3LYP-D4**	273.5	181.6	257.3	246.2	254.5	299.4	303.7	302.0	300.3
**ωB97X-D3(BJ)**	265.3	180.9	248.4	238.8	248.4	289.6	295.9	291.8	291.9
**ωB97X-D4**	271.1	182.1	256.6	244.0	253.8	296.5	302.9	300.5	300.2
**Experimental**	268.2/<br>268.7	170.1/<br>177.4	252.6/<br>254.0	243.3/<br>243.6	252.4/<br>252.5	295.3	301.5/<br>301.6	295.0/<br>296.3/<br>298.0/<br>299.1	294.4

Table S6. Wiberg bond indices for [$GeRu_6(CO)_{18}HI$] obtained in the def2-TZVP basis.

	Ru1–I	Ru5–H	Ge–Ru1	Ge–Ru3	Ge–Ru5	Ru1–Ru2	Ru2–Ru3	Ru3–Ru5	Ru5–Ru6
**TPSS-D3(BJ)**	0.722	0.507	0.490	0.475	0.649	0.412	0.335	0.285	0.328
**TPSS-D4**	0.723	0.506	0.489	0.476	0.647	0.411	0.334	0.284	0.328
**TPSSh-D3(BJ)**	0.722	0.508	0.497	0.480	0.658	0.411	0.337	0.288	0.321
**TPSSh-D4**	0.723	0.507	0.493	0.481	0.655	0.410	0.336	0.288	0.320
**PBE-D3(BJ)**	0.732	0.504	0.483	0.445	0.637	0.399	0.338	0.272	0.347
**PBE-D4**	0.732	0.504	0.485	0.446	0.636	0.399	0.339	0.272	0.348
**PBE0-D3(BJ)**	0.728	0.509	0.505	0.468	0.661	0.405	0.345	0.285	0.329
**PBE0-D4**	0.729	0.508	0.501	0.469	0.659	0.404	0.343	0.284	0.327
**B3LYP-D3(BJ)**	0.679	0.526	0.464	0.462	0.619	0.410	0.362	0.285	0.322

**B3LYP-D4**	0.678	0.526	0.464	0.462	0.618	0.407	0.362	0.284	0.321
**ωB97X-D3(BJ)**	0.720	0.517	0.551	0.556	0.659	0.424	0.341	0.290	0.279
**ωB97X-D4**	0.694	0.508	0.522	0.546	0.660	0.394	0.331	0.291	0.257

Table S7. Computed ^{1}H chemical shift (in ppm) of the [$GeRu_6(CO)_{18}HI$] cluster in the def2-TZVP basis set at the DFT level using various functionals. Relative to tetramethylsilane, computed with the MPSHIFT module of TURBOMOLE.

	**^{1}H (ppm)**
**TPSS-D3(BJ)**	–15.94
**TPSS-D4**	–15.96
**TPSSh-D3(BJ)**	–17.08
**TPSSh-D4**	–17.14
**PBE-D3(BJ)**	–15.28
**PBE-D4**	–15.24
**PBE0-D3(BJ)**	–18.17
**PBE0-D4**	–18.21
**B3LYP-D3(BJ)**	–18.92
**B3LYP-D4**	–18.93
**ωB97X-D3(BJ)**	–19.98
**ωB97X-D4**	–20.86

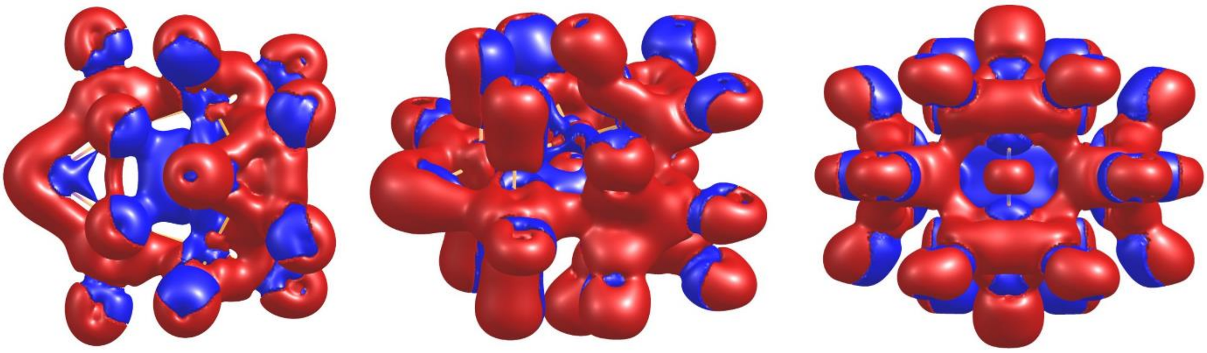

Figure S11. Contour plots of the σ-aromatic system in [$GeRu_6(CO)_{18}HI$] from different perspectives (contour ±0.02 a.u.).

References

[S1] STOE & Cie GmbH: *X-Area, software package for collecting single-crystal or multi-domain crystal data on STOE area-detector diffractometers, for image processing, for the correction and scaling of reflection intensities and for outlier rejection*, Version 1.75, Stoe, Darmstadt 2016.

[S2] X-RED32, *Data Reduction Program*, Version 1.01, Stoe, Darmstadt 2001.

[S3] O. V. Dolomanov, L. J. Bourhis, R. J. Gildea, J. A. K. Howard, H. Puschmann, *J. Appl. Cryst.* **2009**, *42*, 339-341.

[S4] G. M Sheldrick, *Acta Cryst.* **2015**, *C71*, 3-8.

[S5] DIAMOND Version 4.2.2.: *Crystal and Molecular Structure Visualization*. Crystal Impact GbR, Bonn 2016.

[S6] TURBOMOLE V7.6 2021: *A development of University of Karlsruhe and Forschungszentrum Karlsruhe GmbH*, 1989-2007, TURBOMOLE GmbH, since 2007 available from https://www.turbomole.org.

[S7] E. Caldeweyer, C. Bannwarth, S. Grimme *J. Chem. Phys*. **2017**, *147*, 034112.

[S8] a) S. Grimme, J. Antony, S. Ehrlich, H. Krieg, *J. Chem. Phys*. **2010**, *132*, 154104. b) S. Grimme, S. Ehrlich, L. Goerigk, *J. Comput. Chem*. **2011**, **32**, 1456-1465.

[S9] J. Tao, J. P. Perdew, V. N. Staroverov, G. E. Scuseria, *Phys. Rev. Lett*. **2003**, *91*, 146401.

[S10] V. N. Staroverov, G. E. Scuseria, J. Tao, J. P. Perdew, *J. Chem. Phys*. **2003**, *119*, 12129. Erratum: *J. Chem. Phys*. **2004**, *121*, 11507.

[S11] J. P. Perdew, K. Burke, M. Ernzerhof, *Phys. Rev. Lett*. **1996**, *77*, 3865-3868.

[S12] J. P. Perdew, M. Ernzerhof, K. Burke, *J. Chem. Phys*. **1996**, *105*, 9982-9985.

[S13] J.-D. Chai, M. Head-Gordon, *J. Chem. Phys.* **2008**, *128*, 084106.

[S14] P. J. Stephens, F. J. Devlin, C. F. Chabalowski, M. J. Frisch, *J. Phys. Chem*. **1994**, *98*, 11623-11627.

[S15] F. Weigend, R. Ahlrichs, *Phys. Chem. Chem. Phys.* **2005**, *7*, 3297-3305.

[S16] J. Jusélius, D. Sundholm, J. Gauss, *J. Chem. Phys.* **2004**, *121*, 3952-3963.

[S17] K. Reiter, F. Mack, F. Weigend, *J. Chem. Theory Comput.* **2018**, *14*, 191-197.
