# Supplementary material for: [GeRu6(CO)18HI]: A Germanium‐Centered Ruthenium Carbonyl Cluster with Aromatic Ring Current
Source: Adv Sci (Weinh). 2024 Mar 21;11(21):2309043. doi: 10.1002/advs.202309043 (PMC11151064; doi:10.1002/advs.202309043)

ADVANCED SCIENCE
Open Access

Supporting Information

for *Adv. Sci.*, DOI 10.1002/advs.202309043

[$GeRu_6(CO)_{18}HI$]: A Germanium-Centered Ruthenium Carbonyl Cluster with Aromatic Ring Current

*Silke Wolf, Ralf Köppe, Jens Treptow, Wolfram Feuerstein, Jonas Wenzel, Frank Breher, Peter W. Roesky, Florian Weigend, Wim Klopper and Claus Feldmann**

checkCIF/PLATON report

You have not supplied any structure factors. As a result the full set of tests cannot be run.

THIS REPORT IS FOR GUIDANCE ONLY. IF USED AS PART OF A REVIEW PROCEDURE FOR PUBLICATION, IT SHOULD NOT REPLACE THE EXPERTISE OF AN EXPERIENCED CRYSTALLOGRAPHIC REFEREE.

No syntax errors found. CIF dictionary Interpreting this report

Datablock: GeRuCO18HI

```
Bond precision:   O- C = 0.0059 A          Wavelength=1.34143

Cell:             a=8.9282(5)     b=16.5908(8)         c=22.4331(12)
                  alpha=90        beta=112.677(4)      gamma=90
Temperature:      180 K

                 Calculated                      Reported
Volume           3066.0(3)                       3066.0(3)
Space group      P 21/c                          P 21/c
Hall group       -P 2ybc                         -P 2ybc
Moiety formula   C18 H Ge I O18 Ru6              ?
Sum formula      C18 H Ge I O18 Ru6              C18 H Ge I O18 Ru6
Mr               1311.12                         1311.10
Dx,g cm-3        2.840                           2.840
Z                4                               4
Mu (mm-1)        22.456                          22.456
F000             2408.0                          2408.0
F000'            2405.25
h,k,lmax         11,21,29                        11,21,29
Nref             7409                            7046
Tmin,Tmax        0.670,0.799                     0.529,0.654
Tmin'            0.608

Correction method= # Reported T Limits: Tmin=0.529 Tmax=0.654
AbsCorr = MULTI-SCAN

Data completeness= 0.951            Theta(max)= 62.529

                                                       wR2(reflections)=
R(reflections)= 0.0257( 6381)                          0.0686( 7046)

S = 1.056                    Npar= 401
```

The following ALERTS were generated. Each ALERT has the format
test-name_ALERT_alert-type_alert-level.
Click on the hyperlinks for more details of the test.

Alert level B

```
PLAT430_ALERT_2_B Short Inter D...A Contact  O4       ..O13      .       2.81 Ang.
                                                  1+x,y,z  =      1_655 Check
```

Alert level C

```
PLAT029_ALERT_3_C _diffrn_measured_fraction_theta_full value Low .      0.967 Why?
PLAT430_ALERT_2_C Short Inter D...A Contact  O3       ..O4       .       2.86 Ang.
                                                 -1+x,y,z  =      1_455 Check
```

Alert level G

```
ABSMU01_ALERT_1_G  Calculation of _exptl_absorpt_correction_mu
                not performed for this radiation type.
PLAT012_ALERT_1_G N.O.K.   _shelx_res_checksum Found in CIF ......     Please Check
PLAT128_ALERT_4_G Alternate Setting for Input Space Group  P21/c       P21/n Note
PLAT230_ALERT_2_G Hirshfeld Test Diff for    O16      --C16      .        5.3 s.u.
PLAT232_ALERT_2_G Hirshfeld Test Diff (M-X)  I1       --Ru1      .        6.1 s.u.
PLAT232_ALERT_2_G Hirshfeld Test Diff (M-X)  I1       --Ru2      .        8.1 s.u.
PLAT232_ALERT_2_G Hirshfeld Test Diff (M-X)  Ru1      --C1       .        6.6 s.u.
PLAT232_ALERT_2_G Hirshfeld Test Diff (M-X)  Ru1      --C3       .        7.8 s.u.
PLAT232_ALERT_2_G Hirshfeld Test Diff (M-X)  Ru2      --C6       .        5.3 s.u.
PLAT232_ALERT_2_G Hirshfeld Test Diff (M-X)  Ru3      --C8       .        5.1 s.u.
PLAT232_ALERT_2_G Hirshfeld Test Diff (M-X)  Ru4      --C10      .        7.0 s.u.
PLAT232_ALERT_2_G Hirshfeld Test Diff (M-X)  Ru5      --C13      .        5.3 s.u.
PLAT232_ALERT_2_G Hirshfeld Test Diff (M-X)  Ru5      --C15      .        6.0 s.u.
PLAT232_ALERT_2_G Hirshfeld Test Diff (M-X)  Ru6      --C16      .        7.5 s.u.
PLAT303_ALERT_2_G Full Occupancy Atom H         with # Connections       2.00 Check
PLAT883_ALERT_1_G No Info/Value for _atom_sites_solution_primary .     Please Do !
PLAT941_ALERT_3_G Average HKL Measurement Multiplicity ..........        2.5 Low
PLAT965_ALERT_2_G The SHELXL WEIGHT Optimisation has not Converged     Please Check
```

   0 **ALERT level A** = Most likely a serious problem - resolve or explain
   1 **ALERT level B** = A potentially serious problem, consider carefully
   2 **ALERT level C** = Check. Ensure it is not caused by an omission or oversight
  18 **ALERT level G** = General information/check it is not something unexpected

   3 ALERT type 1 CIF construction/syntax error, inconsistent or missing data
  15 ALERT type 2 Indicator that the structure model may be wrong or deficient
   2 ALERT type 3 Indicator that the structure quality may be low
   1 ALERT type 4 Improvement, methodology, query or suggestion
   0 ALERT type 5 Informative message, check

It is advisable to attempt to resolve as many as possible of the alerts in all categories. Often the minor alerts point to easily fixed oversights, errors and omissions in your CIF or refinement strategy, so attention to these fine details can be worthwhile. In order to resolve some of the more serious problems it may be necessary to carry out additional measurements or structure refinements. However, the purpose of your study may justify the reported deviations and the more serious of these should normally be commented upon in the discussion or experimental section of a paper or in the "special_details" fields of the CIF. checkCIF was carefully designed to identify outliers and unusual parameters, but every test has its limitations and alerts that are not important in a particular case may appear. Conversely, the absence of alerts does not guarantee there are no aspects of the results needing attention. It is up to the individual to critically assess their own results and, if necessary, seek expert advice.

Publication of your CIF in IUCr journals

A basic structural check has been run on your CIF. These basic checks will be run on all CIFs submitted for publication in IUCr journals (*Acta Crystallographica*, *Journal of Applied Crystallography*, *Journal of Synchrotron Radiation*); however, if you intend to submit to *Acta Crystallographica Section C* or *E* or *IUCrData*, you should make sure that full publication checks are run on the final version of your CIF prior to submission.

Publication of your CIF in other journals

Please refer to the *Notes for Authors* of the relevant journal for any special instructions relating to CIF submission.

PLATON version of 28/11/2022; check.def file version of 28/11/2022

Datablock GeRuCO18HI - ellipsoid plot

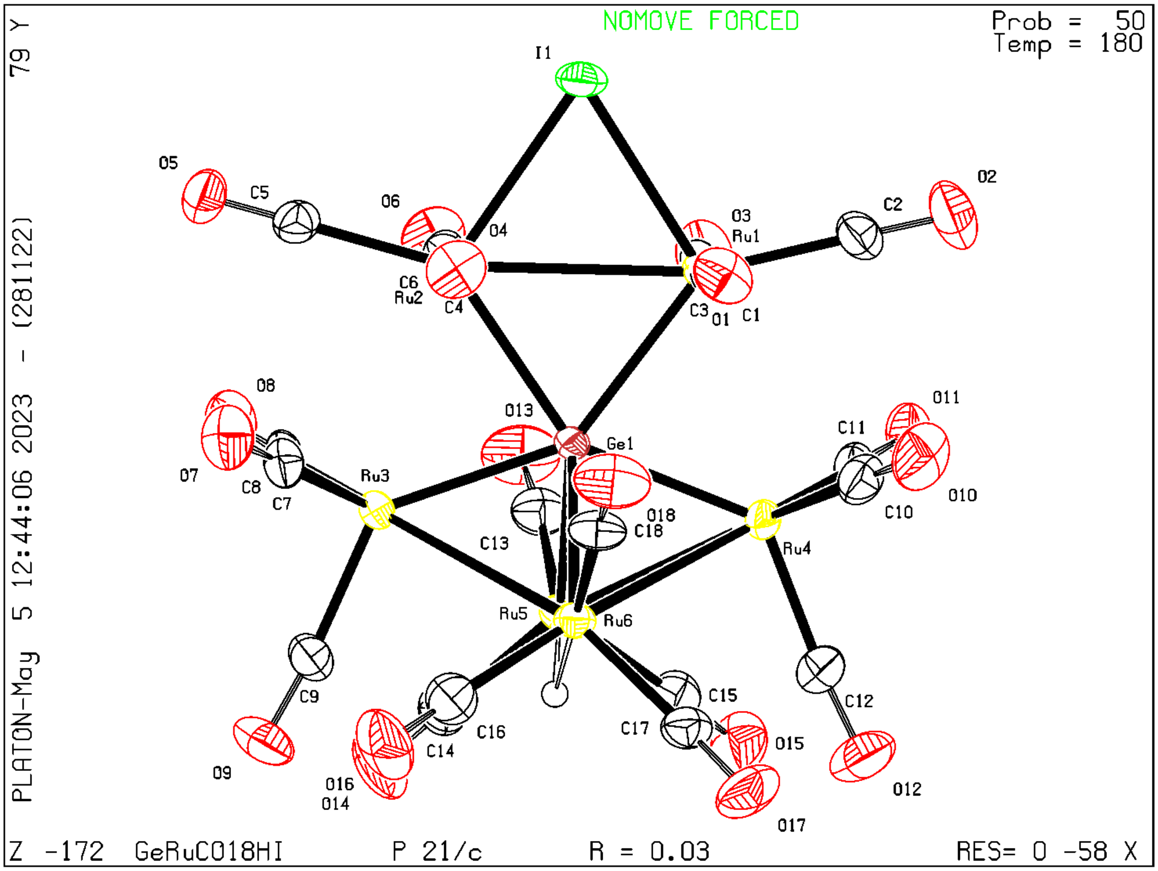

Supplement: Supplementary file 2 — Supporting Information [file ADVS-11-2309043-s001.pdf]
